# Genomic data integration and user-defined sample-set extraction for population variant analysis

**DOI:** 10.1186/s12859-022-04927-0

**Published:** 2022-09-29

**Authors:** Tommaso Alfonsi, Anna Bernasconi, Arif Canakoglu, Marco Masseroli

**Affiliations:** 1 Department of Electronics, Information and Bioengineering, Politecnico di Milano, Via Ponzio 34/5, 20133 Milan, Italy; 2 Present Address: Dipartimento di Anestesia, Rianimazione ed Emergenza-Urgenza, Fondazione IRCCS Ca’ Granda Ospedale Maggiore Policlinico, Policlinico di Milano, Via Francesco Sforza, 35, 20122 Milan, Italy

**Keywords:** Population variant analysis, 1000 Genomes, Human genetic variation, Data integration, Data warehousing, Data wrangling

## Abstract

**Background:**

Population variant analysis is of great importance for gathering insights into the links between human genotype and phenotype. The 1000 Genomes Project established a valuable reference for human genetic variation; however, the integrative use of the corresponding data with other datasets within existing repositories and pipelines is not fully supported. Particularly, there is a pressing need for flexible and fast selection of population partitions based on their variant and metadata-related characteristics.

**Results:**

Here, we target general germline or somatic mutation data sources for their seamless inclusion within an interoperable-format repository, supporting integration among them and with other genomic data, as well as their integrated use within bioinformatic workflows. In addition, we provide VarSum, a data summarization service working on sub-populations of interest selected using filters on population metadata and/or variant characteristics. The service is developed as an optimized computational framework with an Application Programming Interface (API) that can be called from within any existing computing pipeline or programming script. Provided example use cases of biological interest show the relevance, power and ease of use of the API functionalities.

**Conclusions:**

The proposed data integration pipeline and data set extraction and summarization API pave the way for solid computational infrastructures that quickly process cumbersome variation data, and allow biologists and bioinformaticians to easily perform scalable analysis on user-defined partitions of large cohorts from increasingly available genetic variation studies. With the current tendency to large (cross)nation-wide sequencing and variation initiatives, we expect an ever growing need for the kind of computational support hereby proposed.

**Supplementary Information:**

The online version contains supplementary material available at 10.1186/s12859-022-04927-0.

## Background

The introduction of Next-Generation Sequencing (NGS) [1] brought a considerable increase of sequencing throughput, drastically reducing the cost and time for the acquisition of genomic data. Subsequently, the number of genome sequencing initiatives exploded, following the path drawn by the Human Genome Project; the *International HapMap Project* (270 initial samples [2]), the *Cancer Genome Anatomy Project* (7500 samples [3]), the *1000 Genomes Project* [4], and the *100,000 Genomes Project* [5] are only a few examples. Not only the number of datasets but also their quality and volume increased: for instance the 270 genomes of the *International HapMap Project* quickly reached almost 100K units.

In this context, the bioinformatics community is concerned with supporting research advances through the so-called *tertiary analysis* [6], i.e., the interpretation of genomic signals and evaluation of the clinical relevance of genomic features. Inferring knowledge from the collected data, however, remains a challenging task, requiring strong domain knowledge, the integration of multiple heterogeneous datasets, and the implementation of powerful querying tools dedicated to their comprehensive analysis. In particular, data integration is fundamental to interconnect such large amounts of available data—heterogeneous both in the described information and in the representation formats; towards this goal, several solutions have been proposed in the last few years [7–10]. Along with the integration of data, it is essential to make available convenient and efficient instruments to analyze and describe such data by means of meaningful measures (examples are [11–15]).

Considering genomic variation data, the ability to summarize extensive collections of data (possibly originating from multiple sources) and to select samples with specific properties is extremely important. For example, in evolutionary studies, population variation investigations are relevant for finding the predominant DNA characteristics in specific populations; in case-control experiments, they may contribute control populations, whose members are required to meet specific criteria (e.g., genetic mutations, somatic traits, etc.).

Few remarkable solutions to achieve summarization of genomic populations have been proposed (e.g., [16, 17]); yet, at present they exhibit substantial limitations. A major pitfall regards the lack of powerful filtering options and combinations, especially for region data, i.e., on the characteristics of specific positions of the genome. This makes it impossible to select and analyze a genomic population through aggregated measures that satisfy precise requirements on a multitude of aspects. Additionally, the usage of such instruments as components of larger data processing pipelines is hindered by the general absence of helpful programmatic interfaces.

In this work, we intend to overcome some of the shortcomings observed in the currently available solutions, and practically support the mentioned challenges; towards this aim, we developed VarSum, a computational framework with an Application Programming Interface (API) allowing complex queries over an integrated collection of genome variation datasets. Any user-defined combination of metadata filters and fine-grained genome requirements can be applied to identify a population of interest for the user, and retrieve the related dataset. On top of the chosen population, our framework computes different kinds of aggregated statistics, highlighting the most meaningful aspects of the metadata and genomic region data of the population dataset. Finally, we also allow the seamless extraction and downloading of the selected data, to allow processing it with other applications.

VarSum applies to possibly any genomic variation collection of data. Here, we demonstrate its application to the germline variants collected within the *1000 Genomes Project* (1KGP)—currently the biggest public study of population variation data—and to the somatic mutations of *The Cancer Genome Atlas* (TCGA) [18]. We consider the data integration phase an essential preamble to the proper functioning of VarSum on top of multiple data sources. Thus, we describe the integration process of 1KGP into a repository of interoperable genomic resources [19, 20], which already provides the TCGA data in a convenient format. Such integration effort enables users to: (a) locate relevant genomic samples within a large repository of processed data, using the metadata-driven search browser GenoSurf [21], and (b) take advantage of the powerful capabilities of the GenoMetric Query Language (GMQL) [20], by means of a Web server that allows complex queries over the 1KGP genomic samples together with those of TCGA and other genomic catalogues. Moreover, our framework (comprising the integration module and the querying backend and API) meets also the following relevant requirements: (i) ease of integration into existing bioinformatics pipelines; (ii) capability to incorporate arbitrary and new genomic variation data or annotation data sources; (iii) fulfillment of privacy constraints possibly imposed by the organization owning the data—a desirable feature, given the increasing number of private large-scale genome sequencing projects.

**Fig. 1:**
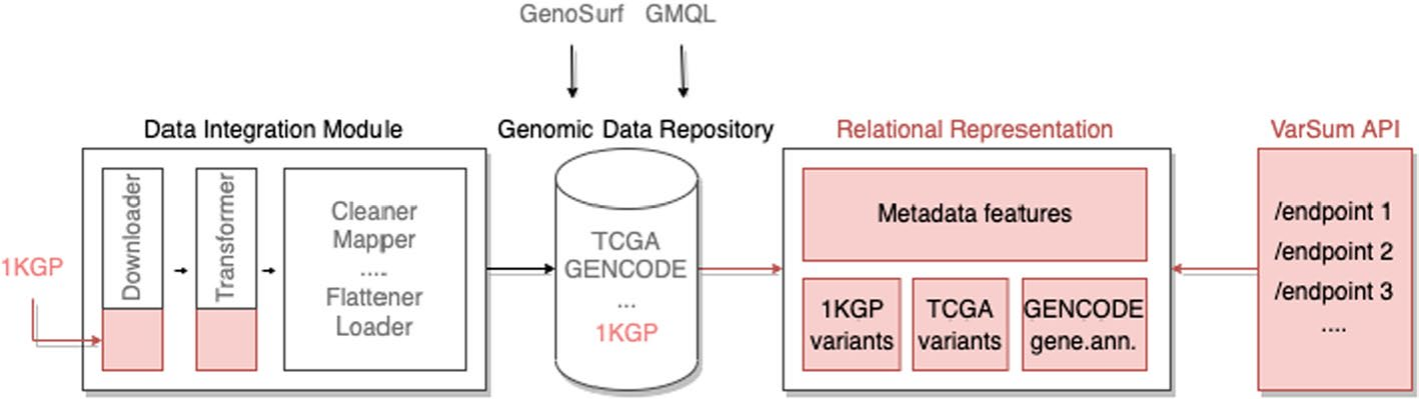
Overview of the proposed framework. Our approach involves developing two source-specific data integration modules for the download and transform stages of our pre-existing data integration framework. Integrating 1KGP into our genomic data repository extends the set of data sources supported by GenoSurf and GMQL, two exploratory and data querying software applications. Then, through its API VarSum provides easy summarization of extensive genomic variation data for user-defined populations; the API queries a novel relational database derived from the genomic data repository for improved efficiency. In red are the novel contributions of this work

Figure 1 illustrates our approach and computational framework: first a data integration module is built to feed a repository of genomic data with the 1000 Genomes Project information; then, the translation of data into a relational representation is implemented, serving as a basis to be queried by the VarSum system through its API. The components that are original of this work are shown in red.

### Related work and current limitations

A number of online tools provide statistics over the genomic variants of large populations, whose data are stored in an associated data source; their characteristics concerning variant analysis are summarized in Table 1.

**Table 1:** Comparison of the features of bioinformatics tools providing aggregated statistics over the variants of a population

	gnomAD	Ensembl	PGG.SNV	EVS	IGIB group
(i) Filters on population by metadata	Limited^a^	–	–	–	–
(ii) Filters on population by region data	–	–	–	–	–
(iii) Gene annotations	$\checkmark$	$\checkmark$	$\checkmark$	$\checkmark$	$\checkmark$
(iv) Quality metrics	$\checkmark$	-	$\checkmark$	$\checkmark$	–
(v) Statistics grouped on metadata	Limited^b^	Limited^c^	Limited^c^	Limited^c^	Limited^c^
(vi) API	–	$\checkmark$	–	–	–

The evaluation concerns (i) the availability of filters on metadata attributes to select a subset of the donors from the dataset, i.e., the population of interest; (ii) the availability of filters to consider only the donors showing particular genomic features (either precise variants or mutated genome regions) distinct from the genomic variant studied in the population; (iii) the possibility to look at the gene annotations and (iv) sequencing quality metrics information concerning a genomic region or variant; (v) the possibility to group the result statistics on the metadata; (vi) the availability of an API

^a^Analysis results are available considering all the donors or one of the seven predefined partitions. On top of the chosen partition, it is also possible to further limit the population to an ethnic group, country (when available), or gender type

^b^The statistics about the variant frequency can be grouped by ethnicity, gender, or geographical origin, if the population has not been already filtered on these attributes. In addition, it is possible to know the age distribution of the donors included in the population and of the variant carriers.

^c^Only for the groups identified by the original sequencing project and/or for the geographical origin of the donors

The Genome Aggregation Database (gnomAD) [16] integrates 68 population surveys of coding variation that have been reprocessed through homogeneous pipelines and jointly variant-called. It is possible to explore the whole dataset or one of its seven pre-defined partitions based on the donors’ metadata. In particular, the “control” dataset partition includes only the healthy donors. Three other partitions are referred to as “non-neuro”, “non-cancer”, and “non-TOPMed”, as they contain all the donors of the dataset except the ones diagnosed with respectively a neurological condition or a cancer pathology, or the ones who are part of the Trans-Omics for Precision Medicine Program [22]. The three remaining partitions include only the samples respectively originating from the Human Genome Diversity Project, or the 1KGP, or belonging to the gnomAD Local Ancestry collection. The population of interest may be further specified by looking at the donors of an ethnic group, country (when available), or gender type. Once the user has chosen the reference population, the interface displays the count of mutated alleles, the total number of alleles, the number of homozygotes, and the allele frequency. The user can obtain the same information also for sub-populations defined by grouping the donors by ethnicity, country (when available) or gender. The analysis of a variant is then enriched with statistics about the read-quality, gene annotations and donors’ age distribution.

Ensembl [17], mostly known for its genome browser, provides additional Web tools to study variants and populations. Similarly to gnomAD, it allows users to compare the frequency of a variant in different populations according to the origin and gender of the samples. In general, the returned statistics are provided by external services. For target variants belonging to the 1KGP, a richer analysis is available, showing the allele frequency, allele count and total alleles in all the 26 populations (and super-groups) of the original study. For these subsets of donors, it reports also the list of samples included in each population and their genotype. Gene annotations are given as well, but read-quality metrics or other information on the donor’s age are not. Ensembl offers programmatic access to this data also via an Application Programming Interface.

PGG.SNV [23] is another important tool for variant analysis; it integrates seventeen genome sequencing projects and provides a complete overview of single variants in terms of total number of alleles, allele count, frequency, gene annotations and read-quality metrics. These values can be scaled to the level of sub-populations of donors, selected according to the study project or the geographical origin.

The Exome Variant Server (EVS) [24] collects somatic mutations from the US country for studying heart, lung, and blood disorders. For any variant available in the database, it can display the allele count, the total allele number and the variant frequency, plus read-quality metrics and gene annotations computed on the whole dataset, or in its partitions. The partitions are only computed based on the geographical origin of the samples.

Finally, it is worth mentioning the contribution given by the Institute of Genomics & Integrative Biology (IGIB) group curating several online tools dedicated to variant analysis, i.e., Al mena [25], IndiGenomes [26] and SAGE [27]. As they share the same front-end design, we discuss them collectively. The type of analysis they provide for a variant includes the frequency, the totally available alleles, the allele count and the number of homozygous alleles for each separated sequencing project. The included datasets are focused on the study of South Asian genomes, which are under-represented in worldwide whole-genome sequencing projects. In addition, their interface provides annotations of the gene located at the variant coordinates.

Our review shows that most current tools provide poor filtering capabilities to choose the population of interest. In this regard, gnomAD stands out by making it possible to choose between one of the seven predefined partitions of the population (see Table 1) and filter on the donors’ gender, ethnicity, or country (when available). Still, it is impossible to focus on analysing a population satisfying arbitrary criteria like multiple countries or ethnic groups in combination with a gender category or specific pathology. The limitations on the selection criteria occur at the metadata level and especially at the region data level. Indeed, despite being possible to collect statistics such as allele count, total alleles and frequency of a variant inside a population, none of the described tools allows specifying a genetic variation as a prerequisite of the population. Population variation data often provides the genotype for each donor, but this information is not employed or reported by any variant analysis tool. As such, it is also impossible to examine only the donors that carry an arbitrarily chosen set of variants inherited from a single parental line. As far as concerns the possibility of comparing the statistics inside sub-populations, we observe that it is not possible to group by a generic combination of metadata provided by the data sources. Even considering gnomAD—which offer the most advanced features in this regard—it does not allow comparing the variant frequency in the sub-populations identified by the country and gender at the same time, nor to group by just the disease.

Many bioinformaticians could benefit from using the above tools as a part of larger software pipelines, eliminating the need for building ad-hoc integrated datasets and simplifying the analysis of large variant sets. Yet, most of the current tools does not support the programmatic use of the supporting integrated datasets, with the only exception being the Ensembl API, which exposes a relevant collection of genomic datasets. Nevertheless, its practical usage is hindered by its complexity, as it requires a deep understanding of the internal data structure and schema, which comprises dozens of tables. With VarSum, we instead propose a simple and powerful API.

## Implementation

### Data integration implementation

The VarSum data integration module extends an already existing software called META-BASE, by implementing a data transformation that is specific for the VCF format, addresses changes in the specific representation of variants, and supports the addition of relevant metadata.

#### Pre-existing META-BASE framework

**Fig. 2:**
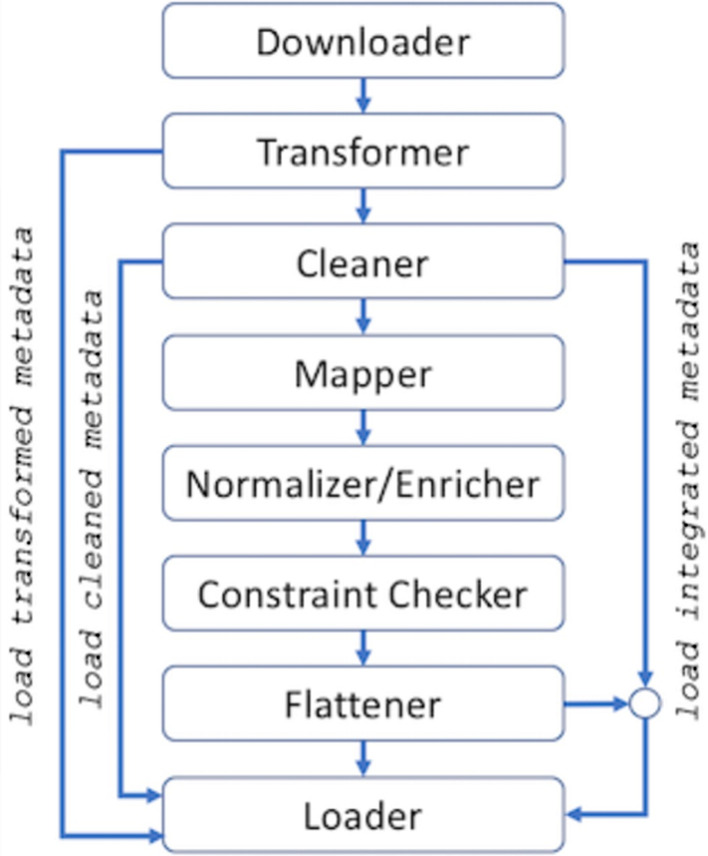
The META-BASE architecture and workflow. Datasets are downloaded from the original source and transformed into a GDM-compliant format. Metadata are cleaned, mapped into the GCM relational integrated data structure, normalized and enriched with related ontological concepts. The homogenized information is checked for correctness, flattened to a file-based data structure and loaded within the META-BASE repository.

Our work is built on top of the META-BASE framework [19], already integrating several heterogeneous genomic data from multiple sources, including ENCODE [28], The Cancer Genome Atlas (via Genomic Data Commons [29]), Roadmap Epigenomics [30], Reference Sequence (RefSeq) [31], and GENCODE [32]. Figure 2 visually summarizes the META-BASE architecture main modules and data integration steps, which are configured through a modular Extensible Markup Language (XML) configuration file (documentation available at [33]). Data are *downloaded* from their original source, and *transformed* into a collection of genomic region data and metadata files according to the Genomic Data Model (GDM) [34]. Metadata files are *cleaned*, by simplifying the output of the transformation (particularly useful in the case of deeply nested data in the original format). Then, their content is *mapped* into the Genomic Conceptual Model (GCM) [35], providing a unifying global schema for metadata of heterogeneous sources. Furthermore, metadata values are *normalized/enriched*, resolving synonyms and linking them to ontological concepts (from well-accepted ontologies such as OBI [36], NCIT [37], and EFO [38]) through a semi-automatic process [39]. An integrity *constraint checker* validates the formal correctness of the obtained metadata values by checking that each attribute holds only acceptable values (e.g., if the species of a sample is “human”, then the assembly value must be one of “hg19”, “GRCh38”, or “hg38”). Finally, metadata are *flattened* back to a file-based data structure in GDM-compliant format and all metadata and region data GDM files are *loaded* into the META-BASE repository of integrated genomic data.

#### From VCF to BED format

While we reused the existing META-BASE infrastructure, the *download* and *transform* phases of the integration workflow required the development of source/format-specific modules. As far as genomic variation data is concerned, the existing META-BASE implementation already provided support for the Mutation Annotation Format (MAF) [40] files and for the integration of public TCGA somatic mutation data [41], which are originally available in MAF format. We extended the META-BASE framework developing new modules for extracting data from Variant Call Format (VCF) [42] files and for the integration of 1KGP data.

**Fig. 3:**
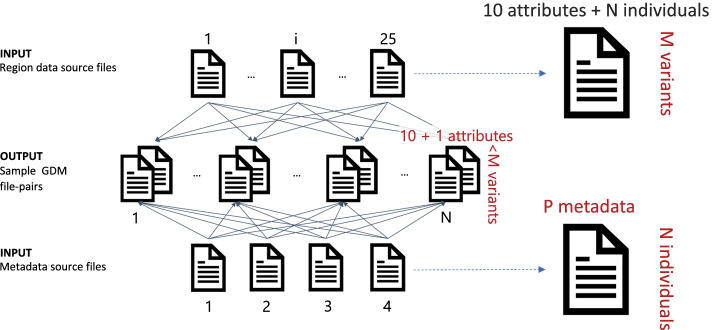
Transformation process for 1000 Genomes Project files. The output sample GDM file-pairs are obtained by: (i) processing the 1KGP big VCF files dedicated to single chromosomes to extract single sample/individual GDM genomic region data files and (ii) distributing the 1KGP metadata information into single sample GDM metadata files

Our extension allows downloading both VCF and metadata files of 1KGP into the local machine. The incremental design allows differentially adding new datasets, or updating old ones; each file local copy is used as a snapshot for detecting changes at the data origin. Datasets downloaded from 1KGP include chromosome-specific VCF files, each containing the variants of all the donors; differently, the chosen target format, based on the GDM (similar to the widely adopted Browser Extensible Data, i.e., BED format [43]), requires a file for each donor, containing all donor’s variants, which in the original source are instead distributed over multiple VCF files. This scenario configures a many-to-many file transformation, exemplified in Fig. 3.

We process all 25 VCF files available in 1KGP, each corresponding to one chromosome and representing a matrix of variants (indicated in rows) that are either present or absent in the donor individuals (as indicated in each corresponding column). In addition, we consider four 1KGP metadata files, organized as matrices where rows represent *N* individuals (identified by the same sample identifier used in region data file columns) and columns contain other information (e.g., regarding the population of the individuals, parent/child relationships, instrument platforms). The transformation algorithm finally builds *N* GDM file-pairs. The number of rows of each GDM genomic region file is typically smaller than *M* (the number of total variants represented in all the VCF input files), as clearly not every individual presents every variant.

#### Changing the variant region format

The transformation process also affects the way in which variants are described within files. Unlike VCF, in BED-like formats, variants become genomic regions requiring both start and stop coordinates:

1. for SNPs or multiple nucleotide polymorphism (MNPs), the stop position is calculated as the available start position plus the length of the substitution;
2. for insertions, the stop position takes the same value of the start position;
3. for deletions, the stop coordinate is equal to the start one plus the length of the deleted sequence.

To allow the alignment with the other genomic features integrated within the META-BASE repository, we employ 0-based coordinates. As a consequence of these two transformations, nucleotide bases that are equivalent in reference and alternative alleles—necessary in VCF to represent indels (insertion-deletion variants) and SVs (structural variants) without ambiguity—become obsolete. Thus, they are removed from both the reference and alternative alleles, and coordinates are adjusted accordingly. Finally, VCF and BED differ in the encoding of multi-allelic variants; these are jointly expressed as a single line combining two or more variants in VCF, while they are independent variants in BED.

Targeting code reusability, we developed a general module that is responsible only for a preliminary transformation, including the separation of multi-allelic variants into multiple bi-allelic variants and the identification of standard VCF attributes. We then implemented a complementary 1KGP-specific module that considers the specificity of the VCF files released by the 1KGP consortium and the International Genome Sampling Resource (IGSR) [44]; it interprets the remaining attributes in these files and is in charge of the following steps:

(i) change of the variant original coordinate system (from 1-based into 0-based);
(ii) simplification of variant definitions used in VCF files, by removing equal bases from reference and alternative alleles (adjusting coordinates appropriately); and
(iii) computation of the variant length and stop coordinate.

The schema of the GDM region files, defining their columns, comprises the 10 attributes characterizing the variants and reported in the Additional file 1; these are the chromosome, the left-end and right-end coordinates, the strand, the original and alternative nucleotides, the mutation length and type information, and two more columns indicating which chromosome copy carries the mutation. An example of translation from VCF to GDM format for genomic region data is provided in Additional file 1.

#### Metadata transformation

Finally, the information included in each input metadata file is assigned to the individual samples (see Fig. 3) and additional metadata about the sample processing and management (e.g., the source of the sample, its local Uniform Resource Identifier - URI, and its format) are appended (see Additional file 2). In order to better support queries on mixed data sources, i.e., over the 1KGP data and the data already present inside the integrated genomic data repository, we computed the value of the ethnicity metadata attribute also for 1KGP data, as it is a common attribute in any sample described through the Genomic Conceptual Model. This was assigned based on the donor’s population as shown in Table 2, and enables several additional queries that make use of the geographical origin to select similar sets of samples between multiple variation data sources.

**Table 2:** Mapping 1KGP population values to ethnicity values

Ethnicity	Pop. Code	Population	In diaspora	Super Population
White	CEU	Utah Residents (CEPH) with Northern/Western Eur. Ancestry	Yes	EUR
	TSI	Toscani in Italia	No	
	FIN	Finnish in Finland	No	
	GBR	British in England and Scotland	No	
	IBS	Iberian Population in Spain	No	
Black or african american	YRI	Yoruba in Ibadan, Nigeria	No	AFR
	LWK	Luhya in Webuye, Kenya	No	
	GWD	Gambian in Western Divisions in the Gambia	No	
	MSL	Mende in Sierra Leone	No	
	ESN	Esan in Nigeria	No	
	ASW	Americans of African Ancestry in SW USA	No	
	ACB	African Caribbeans in Barbados	No	
Latin american	MXL	Mexican Ancestry from Los Angeles USA	No	AMR
	PUR	Puerto Ricans from Puerto Rico	No	
	CLM	Colombians from Medellin, Colombia	No	
	PEL	Peruvians from Lima, Peru	No	
Asian	GIH	Gujarati Indian from Houston, Texas	Yes	SAS
	PJL	Punjabi from Lahore, Pakistan	No	
	BEB	Bengali from Bangladesh	No	
	STU	Sri Lankan Tamil from the UK	Yes	
	ITU	Indian Telugu from the UK	Yes	
	CHB	Han Chinese in Beijing, China	No	EAS
	JPT	Japanese in Tokyo, Japan	No	
	CHS	Southern Han Chinese	No	
	CDX	Chinese Dai in Xishuangbanna, China	No	
	KHV	Kinh in Ho Chi Minh City, Vietnam	No	

The ethnicity value, not specified in the original metadata of 1KGP samples, is assigned based on the available *population* value, thus enabling interoperability with other data sources.

### Data querying implementation

The integration effort described in the previous section is justified by the perspective use of the population variation datasets within an integrative repository and the use of GMQL (or similar) language to flexibly querying the integrated data for any user need. For quickly computing data summarization on the integrated data, however, we deemed a relational database implementation more appropriate. Next, we describe the relational structure that we created to host the integrated data queried by the VarSum API, as well as the articulate architecture of the software application that embeds it, taking care of genomic region data reconciliation, choice of data sources, and query processing.

#### API-targeted data structure

The VCF format was originally proposed within the 1000 Genomes Project Consortium to describe the distribution of population-wide variants. Its data structure is not efficient for selecting a set of donors based on multiple genomic region data and metadata parameters. To improve the performances of such operations, in addition to the GDM format representation, we also built a parallel instance of the 1KGP and other integrated source datasets within a PostgreSQL [45] relational database. Specifically, we employed the datasets resulting from the genomic data integration phase : the new database is created during the Mapper step and finalized during the Constraint Checker step of the META-BASE framework (see Fig. 2). Such a database holds one table for each GCM entity (i.e., Item, Replicate, BioSample, Donor, ExperimentType, Dataset, CaseStudy, and Project) for the metadata of all sources collectively. Furthermore, to load the region data inside the database, we implemented a Python script that leverages the output of the META-BASE Flattener stage. The script receives in input the GDM region data files, the list of region attributes to load, and the name of the output region data table. By parsing the list, it loads every region data attribute to the corresponding column of the target table.

**Fig. 4:**
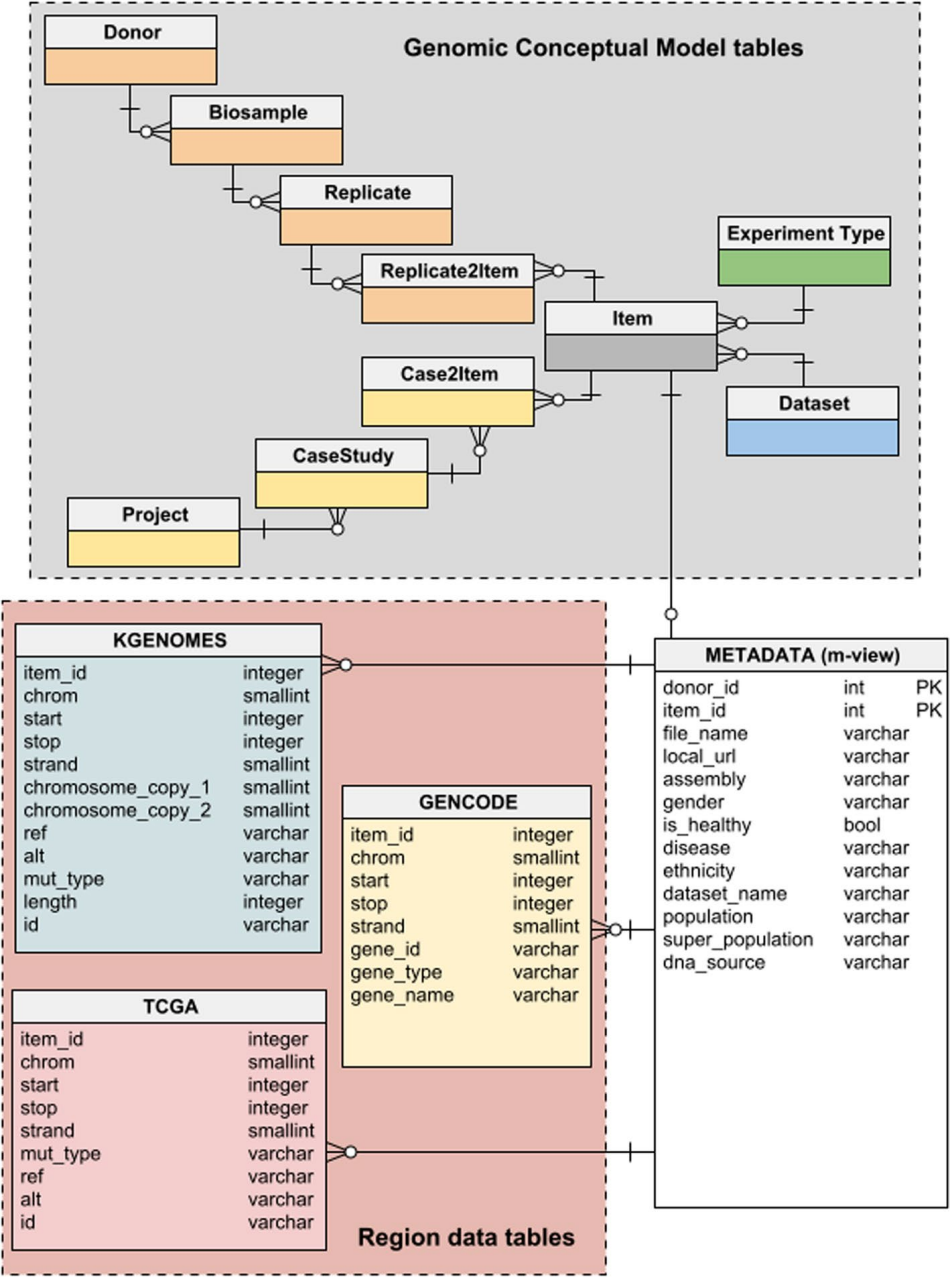
Relational data model for VarSum. It is composed by a table for the region data of each data source (e.g., 1000 Genomes Project - KGENOMES, TCGA, GENCODE) and one materialized view (METADATA) providing fast access to the metadata of all sources. The materialized view is obtained as a selection of the most important attributes for VarSum from those comprehensively available in the tables of the database [21] based on the Genomic Conceptual Model [35], here represented only through their names

The script has been designed to be general enough to load different types of GDM datasets; indeed, we applied it for loading the newly generated GDM datasets of 1KGP variants, as well as those of the TCGA somatic mutations and GENCODE gene annotations, resulting in a single database (illustrated in Fig. 4). For performance reasons, a materialized view has been created to pre-compute the join of different entities of the GCM schema. Note that the region data tables generated in this stage include only a part of the region data attributes originally integrated in our main GDM repository and usable with GMQL; these attributes are indeed the most relevant for the purpose and functions exposed by VarSum.

#### Server software architecture

**Fig. 5:**
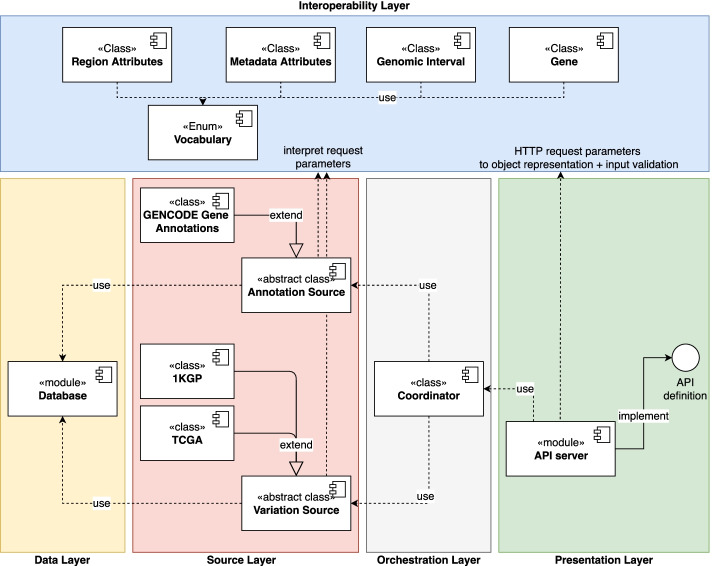
Overview of the server software architecture of VarSum

The VarSum client-server software architecture, implemented with Python programming language [46], is accessible to users through a flexible API. As shown in Fig. 5, the server side is structured in five separate layers, with limited inter-layer interactions. The Presentation layer directly interacts with the user, by exposing the API endpoints. The execution of the requests is delegated to the Orchestration layer, specifically to its *Coordinator* module; through a standardized interface, this interacts with the data sources available in the Source layer for executing the requests. The Data layer manages the connection with the database holding the variants of the 1000 Genomes and TCGA projects, and the GENCODE gene annotations. Finally, the Interoperability layer provides elements that facilitate the communication between the other layers; for example, it explicitly defines the concepts of genomic variant and gene, and all the metadata and genomic region attributes recognised by the API.

#### Genomic region data value reconciliation

The API-targeted database inherits the metadata homogenization effort performed by the mapping and enrichment phases of the META-BASE framework. Genomic region data attributes, instead, are only uniform in the four mandatory fields (i.e., chromosome, strand, start and stop coordinates). Depending on the represented data type, every source may report schemata with additional attributes, with diverse syntax and semantics, even using synonyms and homonyms, for their names and values. Within our specific focus on genomic variation data, we handled issues involving name reconciliation and value transformation/merging.

Moreover, we defined a minimal set of categories of region data attributes, considered essential for any variant definition. These extend the GDM mandatory genomic region attributes with variation data information about the reference and alternative alleles, a variant-ID referencing an external database (e.g., dbSNP), and the variant type. Each attribute is represented in the *Vocabulary* class of the server software Interoperability layer (Fig. 5), as a general information category to which other classes can refer. The main advantage of this representation is the possibility to programmatically formulate data-agnostic queries using such categories. These queries are then translated into concrete Structured Query Language (SQL) queries by each *Variation Source* class in the Source layer, according to the specificity of the source table. In case multiple sources are involved in answering a request, multiple tables are queried; the outcome of every query is expressed using the *Vocabulary* attributes, which simplifies the merging of results. As a consequence, the architecture allows to incorporate into the database new genomic variation data or annotation data sources, without constraining the schema of the region data tables.

#### Selection of the eligible data sources for answering a request

The *Variation Source* and *Annotation Source* classes of the server software Source layer employ the items of the *Vocabulary* class to communicate the maximum filtering capability over a source. For example, 1KGP data include the chromatid of the variants (stored in the chromosome_copy_1 and chromosome_copy_2 attributes of the KGENOMES table), making it possible to select variants occurring on the same chromatid. The same information is not available in TCGA; then, only the 1KGP source may be used to answer queries asking for variants on same/opposite chromatids. Such possibility is reflected in the software because the classes *1KGP* and *TCGA* of the Source layer own different subsets of the information categories described in the *Vocabulary*. When an API endpoint is called, the *Coordinator* class determines the data sources that are eligible to answer the request (i.e., the ones that declare a set of available filters greater or equal to the one contained in the request).

#### Query computation

When the VarSum API receives a request, this is interpreted by the *API Server* class, which performs input validation and delegates the computation of the result to the *Coordinator* class. The *Coordinator* identifies the data sources that are eligible for satisfying the filters indicated in the request, and asks the corresponding software classes to perform a query with the abstractions provided by the *Vocabulary* class. The query is translated into an actual SQL query by each of the *Variation Source* or *Annotation Source* classes involved, to account for the unique characteristics of the corresponding source region data. The formulated queries are executed when the *Coordinator* class retrieves the control; it assembles the received SQL instructions into a single query and finally computes the result. The outcome is then returned to the *API Server* class, which formats the result into a JavaScript Object Notation (JSON [47]) encoded table or list.

VarSum supports both (1) simple “exploratory-based” requests, returning information already explicit in the source and whose implementation is trivial; and (2) “aggregated measures” requests, computing their results as a transformation of the source data. In the following, we elaborate on the query computation for the main types of requests of the second kind: /donor_grouping, /variant_grouping, /most_common_variants, and /rarest_variants.

##### Aggregation of donors

The endpoint /donor_grouping considers in input the characteristics of an arbitrary population and a grouping scheme; it finds the donors satisfying the requirements and counts the size of each group of donors. This request is satisfied by the *Coordinator* class using an SQL instruction in the form reported in Listing 1, where ATT_1 and ATT_2 are generic placeholders for the metadata attributes (one or more), whose values are used to compute the groups. The FROM clause at lines 2–12 includes the definition of the data describing the population of interest, collected from the data sources; if there is more than one data source, the populations selected by each *Variation Source* class are merged together via a UNION clause (line 7), eliminating the duplicates. Comments at lines 5 and 10 are placeholders for the query received by the involved *Variation Source* classes, whose purpose is to select the population according to the requirements. Their formulation can be very different as they are specific for each source.
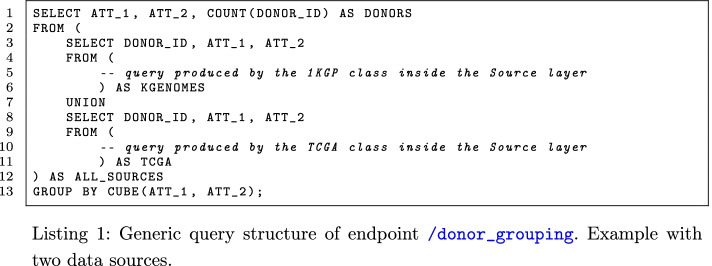


##### Variant frequency inside a population

The endpoint /variant_grouping computes the frequency of a variant inside a population. The *Coordinator* class issues a query as the one reported in Listing 2, where ATT_1 represents a generic metadata attribute (or a list of them) on which the population is grouped. The population is the union of what has been selected by each *Variation Source* class according to the requirements given in the request; every *Variation Source* participating to the request must return the *DONOR_ID*, the *GENDER*, the value of ATT_1, and how many times the variant is present—between 0 and 2—for each donor in the population. The last two attributes are necessary for computing the frequency of a variant within the population. Indeed, the frequency value depends on the number of occurrences of the variant (*OCCURRENCE_OF_TARGET_VARIANT* in the example) and the total number of alleles. However, the assembly used affects the location of pseudoautosomal regions in sexual chromosomes; so, also the location of the variant (generically indicated as *CHR* and *POS* in Listing 2) as well as the gender become important to calculate the number of total alleles. The frequency formula is implemented in and computed by the functions *MUTATION_FREQUENCY_HG19* and *MUTATION_FREQUENCY_GRCH38* (depending on the reference genome indicated in the request) scripted in the PostgreSQL server.
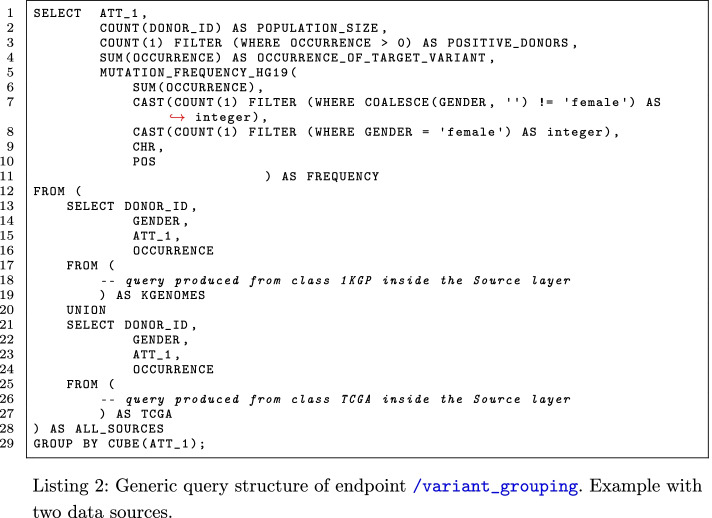


##### Ranking of variants

The endpoints /most_common_variants and /rarest_variants are based on a query similar to the one shown in Listing 2. The ranking of variants requires the eligible data sources—and so the corresponding *Variation Source* classes—to return a query providing the list of mutations occurring within any genome of the population selected in the request. Every mutation must be indicated through the attributes *CHROM*, *POS*, *REF* and *ALT*, and annotated with the number of donors exhibiting the mutation, the total number of alleles in the population, and the number of occurrences of the variant inside the population. The *Coordinator* class embeds the queries provided by the sources into an outer query, which groups the mutations by their coordinates, reference and alternative alleles, and sums the numeric variables in each group; for each group, i.e., for each variant, it then computes the frequency as the ratio of occurrences over the total number of alleles.

## Results

### Integration of the 1000 Genomes Project population variation data

Two datasets of 1KGP data are publicly available. The oldest one, dated 2015, represents the original outcome of the project as joint work of the 1000 Genome Project Consortium and the Structural Variation Analysis Group. It comprises single nucleotide polymorphisms (SNPs), indels and structural variants from the DNA or ribosomal DNA (rDNA) of 2535 samples/donors, whose genomes were aligned to the hg19 reference human assembly. The International Genome Sampling Resource , which was established to expand and maintain the project after its conclusion, realigned these variants to the GRCh38 reference human genome two years later and added those of 13 samples/donors; however, such more recent dataset only includes SNPs and bi-allelic indels. The two datasets are stored at specific locations within a 1KGP FTP repository^1^. Each dataset contains a collection of 23 VCF files, one for each human chromosome from 1 to 22, plus chromosomes X; the hg19 dataset includes two more VCF files for the chromosomes Y and MT (i.e., mitochondrial). Each file represents a big set of variants that may or may not be present in each sample. Samples belong to control population individuals, i.e., healthy individuals’ genomes compared against the reference genome. Samples are further described by 4 single metadata files containing different metadata types^2^.

By means of the META-BASE framework and the new software modules we developed for its extension, we generated two new GDM datasets of 1KGP data:

- HG19_1000GENOMES_2020_01, containing the full 1KGP outcome data aligned to the hg19 genome assembly;
- GRCh38_1000GENOMES_2020_01, including the 1KGP biallelic SNP and indel variants aligned to the GRCh38 genome assembly.

These are, to date, considered the most updated and stable versions of the 1KGP project. Details concerning the number of files transformed, the size of the datasets and the execution time for the entire process (including all the stages of the META-BASE pipeline) are reported in Table 3.

**Table 3:** Statistics of the 1KGP input and output datasets

	Dataset	Region files	Metadata files	Size (.gz compressed)	Size (uncompressed)
Input	hg19	25	4	17 GB	796 GB
	GRCh38	23	4	13 GB	752 GB

	Dataset	Region files	Metadata files	Size	Execution time
Output	hg19	2535	2535	1.5 TB	217 h 7 min 40 s
	GRCh38	2548	2548	1.1 TB	187 h 59 min 45 s

The transformation process was executed on a server machine equipped with an Intel(R) Xeon(R) CPU E5-2660 v4, 378 GB of RAM and an array of mechanical hard disk drives of a total size of 48 TB. The process is mainly input/output bound; so, despite the parallel implementation using 15 processors out of 28, it took a considerable amount of time; the difference between the processing time of the two datasets is a consequence of the different content of the two datasets: the GRCh38 dataset released by IGSR includes SNPs and bi-allelic variants, but lacks longer structural variants, resulting in a smaller number of variants and reduced complexity of the transformation overall.

The two generated datasets are provided, together with several other genomic datasets previously available, in the GMQL system through its Web interface [54] or APIs (RESTful, Python and R/Bioconductor) as part of a large integrated genomic repository documented in [20]. They are also searchable in GenoSurf [21, 55], where both the original metadata and the transfomed metadata values—as resulting from the integration process—can be used for filtering.

#### Already integrated sources: TCGA and GENCODE

The integration of 1KGP data inside our genomic data repository allows using the 1KGP data together with the datasets that were there previously integrated. For instance, a user may wish to combine multiple types of features and compare the 1KGP variants with other genomic regions, either variants or annotations. To exemplify this possibility, we selected another source of variation data from our repository, i.e., The Cancer Genome Atlas [18]. Similarly to the 1KGP, TCGA somatic mutations are organized into two datasets indicated in our repository respectively as HG19_TCGA_dnaseq, containing the variants of 6,914 samples aligned on the hg19 assembly, and GRCh38_TCGA_somatic_mutation_masked_2019_10 for the variants of 10,187 samples aligned on the GRCh38 assembly.

Our integration of the two sources allows performing identical queries on both, comparing the results, and easily extracting their data through any of the discussed interfaces: the VarSum API here presented, GMQL, or GenoSurf. Note that not all region/metadata attributes in 1KGP are available in TCGA. Available filters can be inspected in Table 4.

**Table 4:** Filtering capability for TCGA and 1KGP

	Filter samples on	Availability in source	
		TCGA	1KGP
Metadata	Gender	x	x
	Ethnicity	x	x
	Population		x
	Super population		x
	Health status	x	x
	Disease	x	x
	Assembly	x	x
	DNA source (i.e., LCL/blood)		x
Region data	Presence of a specific variant/multiple variants	x	x
	Absence of a specific variant/multiple variants	x	x
	Presence of any variant inside a specific genomic region	x	x
	Presence of two or more specific variants on the same chrom. copy		x
	Presence of two specific variants on opposite chrom. copies		x
	Having any germline variant		x
	Having any somatic variant	x	

In VarSum, we provide the annotations imported from the HG19_ANNOTATION_GENCODE and GRCH38_ANNOTATION_GENCODE datasets, both already integrated into our genomic repository and available through its interfaces. The datasets originate from the GENCODE initiative [32], and describe the start and stop positions of genes, respectively aligned on hg19 and GRCh38 human assemblies.

While VarSum currently includes only 1KGP, TCGA and GENCODE data sources, several additional data sources can be easily integrated next, undergoing a necessary prior integration within the META-BASE repository, if not already performed.

### VarSum API

The VarSum interface is implemented as a RESTful API, to facilitate the integration inside already existing pipelines. Its full corpus of the API POST and GET endpoints is listed in Table 5.

**Table 5:** Endpoints available in the VarSum API

HTTP method	Function	Endpoint
POST	Measure-based	http://www.gmql.eu/popstudy/api/donor_grouping
POST	Measure-based	http://www.gmql.eu/popstudy/api/variant_grouping
POST	Measure-based	http://www.gmql.eu/popstudy/api/most_common_variants
POST	Measure-based	http://www.gmql.eu/popstudy/api/rarest_variants
GET	Exploratory	http://www.gmql.eu/popstudy/api/values
POST	Exploratory	http://www.gmql.eu/popstudy/api/annotate
POST	Exploratory	http://www.gmql.eu/popstudy/api/variants_in_region
POST	Exploratory	http://www.gmql.eu/popstudy/api/download_donors

The first four endpoints (/donor_grouping, /variant_grouping, /most_common_variants, /rarest_variants) implement the main data querying functionalities of the API; for a group (i.e., population) of individuals, they respectively return the following measures:

- the donors’ counts for each partition of the population;
- the frequency of a variant inside each partition of the population;
- the most frequent variants in the population;
- the rarest variants in the population.

Query parameters can be set as JSON elements in the POST payload of the request to filter the requested population based on donors’ metadata and exhibited variants.

Four additional endpoints (/values, /annotate, /variants_in_region, /download_donors) are provided for data exploration, specifically to:

- extract all distinct values of a metadata attribute;
- annotate a genomic region by means of GENCODE gene annotations;
- list the variants appearing in a given genomic region of a population;
- download the metadata and genomic region data of the population of interest in GDM format.

All endpoints return responses in the form of JSON files. Endpoints that perform aggregation (i.e., /donor_grouping and /variant_grouping) return a data cube showing counts or frequencies relative to one or many combined properties within the population. Other endpoints return a table, or a simple list. A call to VarSum only requires three Python instructions, as shown in Listing 3.
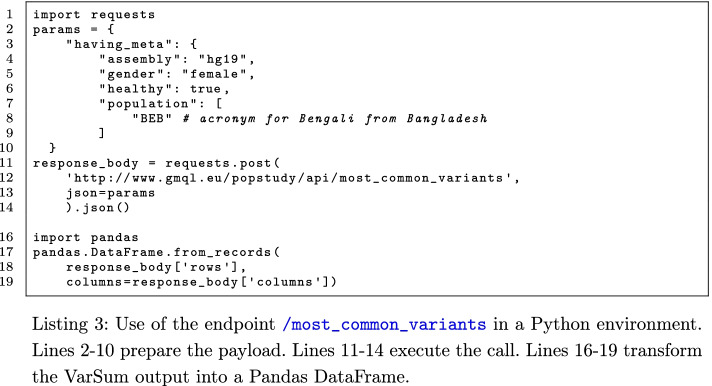


The API endpoints have self-descriptive names and reusable body parameters. Most endpoints share the same request body schema definition. For example, the request body *params* in lines 2–10 of Listing 3 is a valid input for the endpoints /most_common_variants and /rarest_variants; but it becomes a valid input also for the endpoint /donor_grouping once we add the attribute group_by. Furthermore, by adding a target_variant, the request body can be used also for calling the endpoint /variant_grouping. A list of the valid assignments of metadata attributes is available at the endpoint /values. Instead, region attributes can be specified in multiple ways: genomic regions can be described as named annotations (e.g., the gene name *IDH1*), or as arbitrary intervals through the information about the chromosome, start and stop; variants can be identified by means of the values chromosome, start, and reference and alternative alleles, or also by the mutation ID as assigned by dbSNP [56]. Lines 16–19 of Listing 3 show how the response output may be transformed into a Pandas DataFrame [57], for further processing.

A complete documentation of the endpoints is available at [58]; under each endpoint, we define the structure of the allowed input parameters and provide a few examples. Users can try the API functions directly on the documentation page with ready-made examples, or custom parameters. Additionally, we provide examples of use of the API in form of IPython Notebooks [59] and Google’s Colab Notebooks [60] inside the /demo directory of the project repository [61]; two of them, (*UC A - identification of mutations involved in development of brain lower grade glioma* and *UC B - differential mutation analysis to unveil cancer genes*) are demonstrative applications explained also in the next section, while the other four are useful examples to familiarise with the body parameters and the API endpoints.

### Use cases

In this section we show example cases of use of the results of our effort. The first use case demonstrates how 1KGP datasets can interoperate with other sources in the META-BASE repository, by means of the GMQL query language. The second use case employs a simple exploratory API call to evaluate donors’ populations. The last two use cases provide instead more elaborate examples of how to compose several calls to the VarSum API to perform advanced analyses.

#### Integrative queries with GMQL

The META-BASE repository is accessible through the GMQL web interface [54], where datasets of several integrated genomic data sources are available. GMQL provides cloud computation supporting queries over several samples in parallel, taking into account genomic region positions and distances.

As an example of possible integrative queries, in Listing 4 we take advantage of the previous integration of ENCODE ChIP-seq data into the META-BASE repository, documented in [35]; we present a GMQL query that allows annotating the genomic variants of the 1KGP dataset with the H3K4me1 histonic modification regions of protein H3 for the human colon cancer cell line HCT116 (line 1). To demonstrate its use on a small data set, we selected only the female donors from Tuscany region of Italy (line 2). Then, by employing operators of the GMQL language, we created a unique dataset unifying both initially selected data sets (line 3), and only extracted those genomic regions that are covered by at least two initially selected variant and H3K4me1 regions (line 4), finally preserving only metadata relevant to the example (i.e., population, donor_id, and gender) (line 5). The result is eventually materialized and can be downloaded for further inspection.
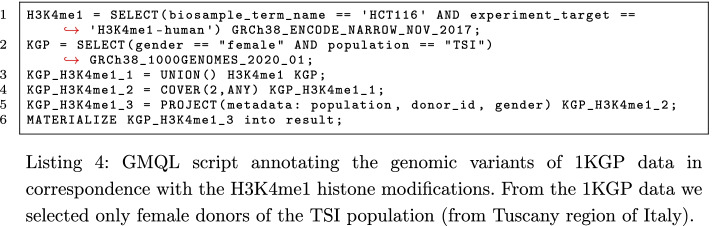


#### Extraction of a donors’ population

In this use case, we show how it is possible to choose a population of donors that satisfies precise requirements, also regarding genomic variants. We can start by inspecting the metadata properties of a dataset using the VarSum API endpoint /donor_grouping/donor_grouping, for example with the request body in Listing 5.
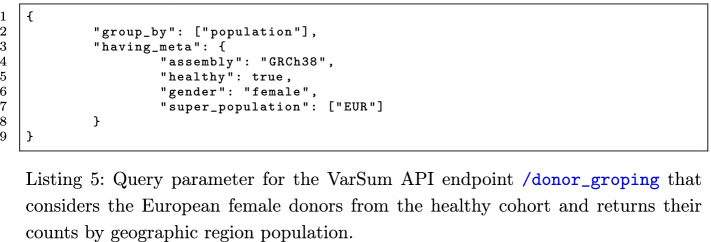


If we are interested only in the donors whose genome shows any variation in one or more genomic regions (e.g., in the miRNA gene MIR4657), we can specify them as a requirement, as in Listing 6.
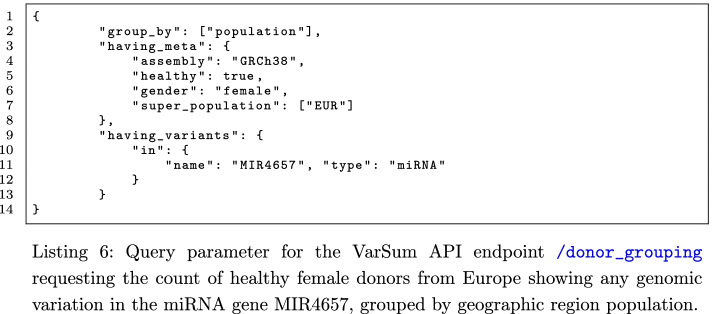


Once we are satisfied with the selected population, we can perform other queries to explore further the population through the endpoints of the VarSum API (e.g., to know the most common variants), or also export the population dataset and analyse it using other arbitrary tools. To download a population data, we make available the endpoint /download_donors, which takes as argument the same input of the endpoints /donor_grouping, /variant_grouping, /most_common_variants, or /rarest_variants; therefore, to download the current population, we can use the input of previous Listing 5 or Listing 6. The downloaded files consist in one “.gdm” and one “.gdm.meta” file for each donor, describing respectively the genomic region data and the metadata of the donor.

#### Identification of mutations involved in the development of brain lower grade glioma

Here, we illustrate how to extract a disease population of interest from the integrated data, and identify: (i) the variants that are most likely involved in the disease, and ii) the affected genes. In this use case, we use the TCGA data available for use in VarSum thanks to its prior integration inside the META-BASE repository. Focusing on *brain lower grade glioma*, Listing 7 reports the sequence of calls made to the VarSum API for this study. First, we call the /most_common_variants endpoint with the payload shown in lines 2-11 to find the most common somatic variants in patients affected by the considered disease. The service returns the mutation 2:208248387:C:T (referred to as TM1 in the following) as the top one, occurring in almost 70% of patients.
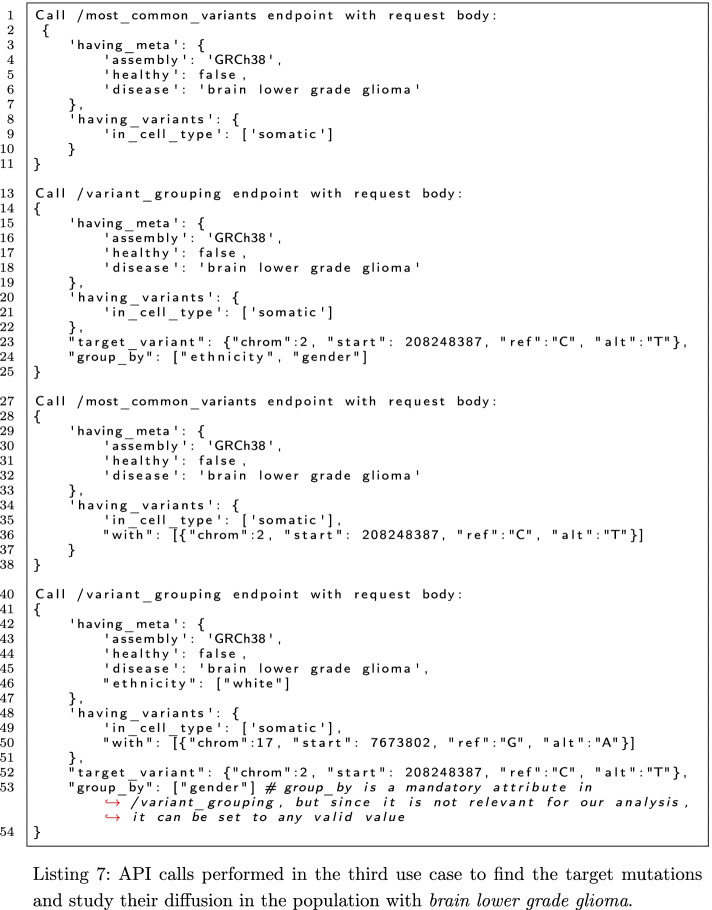


Such frequency has been calculated in the entire population of donors with the disease, but we can also look at the frequency in the sub-populations by calling the /variant_grouping endpoint with the parameters indicated in lines 14-25 of Listing 7. The response reveals that out of all patients affected by *brain lower grade glioma* and with the TM1 variant (509), most (468—92%) are of white ethnicity. However, the population size in other ethnic groups is too small to make considerations about the population prevalence of the genetic trait or the disease; the under-representation of other ethnic groups in the dataset (white donors cover 75% of the patients analysed in TCGA) is the most likely cause of this bias. Finally, the result also shows almost identical distribution of cases between males and females.
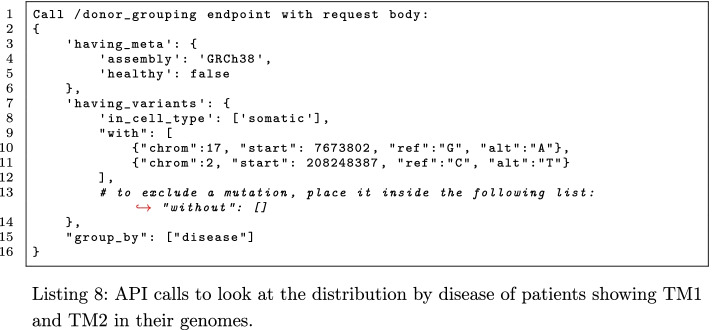


We identified the next target mutation (TM2 in the following) by calling the endpoint /most_common_variants as in lines 28-38 of Listing 7. The result tells that TM2 is 17:7673802:G:A and occurs in almost 10% of the *brain lower grade glioma* patients with TM1. While the frequency of TM2 is not so high in such donors, the opposite holds: by calling /variant_grouping with the payload shown in lines 41-54 of Listing 7, we observe a much higher frequency of TM1 in the white ethnicity donors having TM2 and *brain lower grade glioma*: almost 80%. This result suggests that TM1 and TM2 are the mutations that are most likely involved in the development of such disease.

**Fig. 6:**
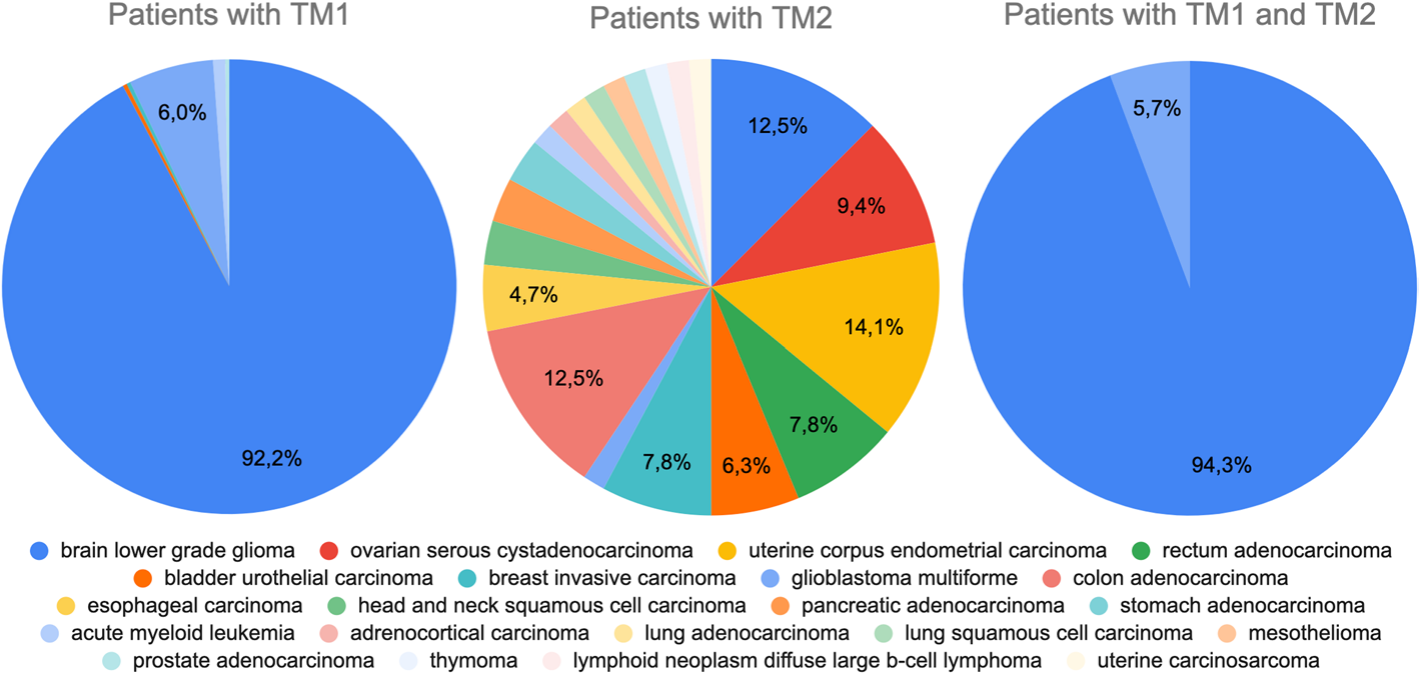
Comparison of the pathologies of donors with only TM1, or only TM2, or both TM1 and TM2 mutations. TM1 is found in 6 groups of patients corresponding to widely different pathologies, including *brain lower grade glioma*, *bladder urothelial carcinoma*, *breast invasive carcinoma*, *glioblastoma multiforme*, *acute myeloid leukemia*, and *prostate adenocarcinoma*. The co-presence of both TM1 and TM2 mutations reduces the number and types of associated pathologies to only 2 (both brain tumors), and increases of 2.1% the likelihood of correctly detecting the *brain lower grade glioma*

To strengthen our findings, we further tested the distribution by disease of the donors having TM1 and TM2, using the endpoint /donor_grouping with the request parameter at lines 2–16 of Listing 8; the call can be repeated moving alternatively one of the two mutations inside a “without” list of variants in the “having_variants” group to obtain the distribution of donors having TM1 but not TM2, or vice versa. The results are reported in Fig. 6.

Finally, by querying the endpoint /annotate first with the parameters shown at lines 2-8 and then with those at lines 9-15 of Listing 9, we observe that the genes IDH1 and TP53 are mutated respectively by the variants TM1 and TM2.
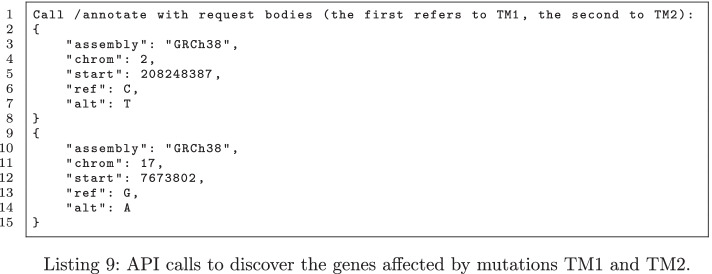


This finding is confirmed in the literature (Ichimura et al. [62]), where the combination of IDH1 and TP53 mutations is found to be a frequent and early change in the majority of secondary glioblastomas, a more severe type of brain tumor originating from lower grade glioma.

#### Differential variant analysis to unveil cancer genes

In [63], Przytycki and Singh proposed a technique (named DiffMut) to identify genes that are likely involved in a given disease, based on the comparison of their somatic mutations and germline variants. Taking advantage of the VarSum functionalities, that relevant technique can be easily implemented using VarSum, only assuming a few simplifications to the original method (discussed in the following). Thus, leveraging on the performed integration of genome variation data from different sources, it can be applied on the aggregated data (in this use case we use GENCODE gene annotations, TCGA and 1KGP mutations and variants) for different studies of biological and clinical interest. Here, we show how to do it, focusing on finding genes that are involved in *skin cutaneous melanoma*.

The adopted general strategy can be summarized as follows:

1. Select a set of candidate genes G
2. Separately for a group of healthy donors (healthy cohort) and a group of patients affected by the considered disease (tumor cohort):
  - Assign a score $S_{i}$ to each gene $G_{i}$ for a cohort: being $V_i^c$ the set of variants present in the cohort *c* and falling in the genomic region of $G_{i}$, and $count^c(v)$ the number of times a variant *v* occurs in the donors of the cohort *c*, compute the score as $S_i^c = \frac{\sum _{v\in V_i^c} count^c(v)}{size(c)}$
  - Rank-normalize the gene scores in a cohort, by assigning value 1.0 to the gene with the highest score and lower positive values to the other genes, proportionally to their score $S_{i}$ (this normalized value is a measure of how much a gene is likely to mutate within the considered cohort)
3. Compare the rank-normalized scores to find the genes where they differ the most between the tumor and the healthy cohorts.

Compared to the DiffMut technique that analyses the single patient’s genomes, in VarSum we make use only of aggregated data; therefore, the need to adapt the method. The original technique counts the variants falling in each candidate gene for every donor in the cohort under consideration, thus obtaining a distribution of variant counts for every gene. Then, to estimate the role of the gene and its alteration in the tumorigenic event, DiffMut computes the difference between the two cohort distributions for the gene, using a novel measure called unidirectional Earth Mover’s Difference (uEMD). We replaced the mutational profile of each gene (a distribution over donors) with the gene score $S_{i}$ previously illustrated, and consequently the uEMD with the difference of the gene scores.

For computing the gene scores, we first extract the list of variants in a gene for the tumor and healthy cohorts; this can be done by calling the /variants_in_region endpoint of the VarSum API with the gene name as a parameter (see Listing 10 for the tumor cohort), repeating the operation for each gene in the candidate list.
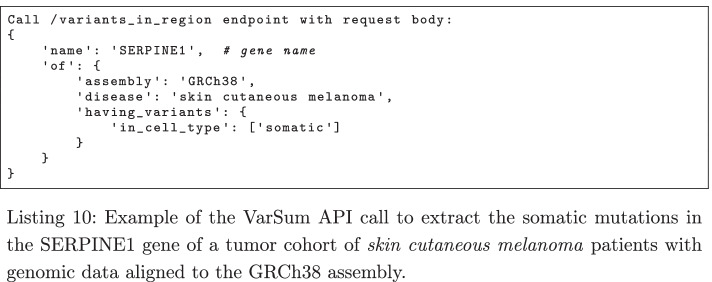


The next step to compute the gene scores involves extracting and counting the occurrences of a specific gene variant, and knowing the size of the cohort. We can obtain both information by calling the VarSum API endpoint /variant_grouping as in Listing 11; the interesting values for our analysis are contained in the output columns OCCURRENCE_OF_TARGET_VARIANT and POPULATION_SIZE, which is a constant of the cohort.
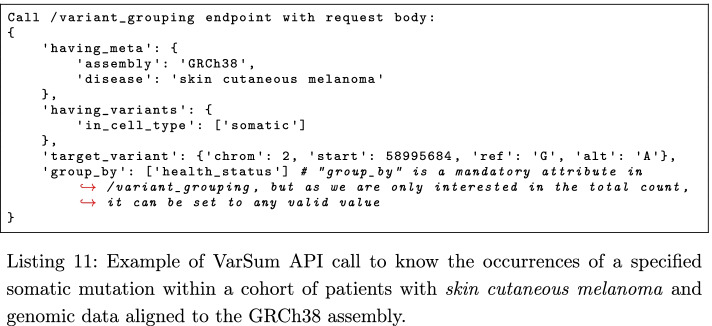


**Fig. 7:**
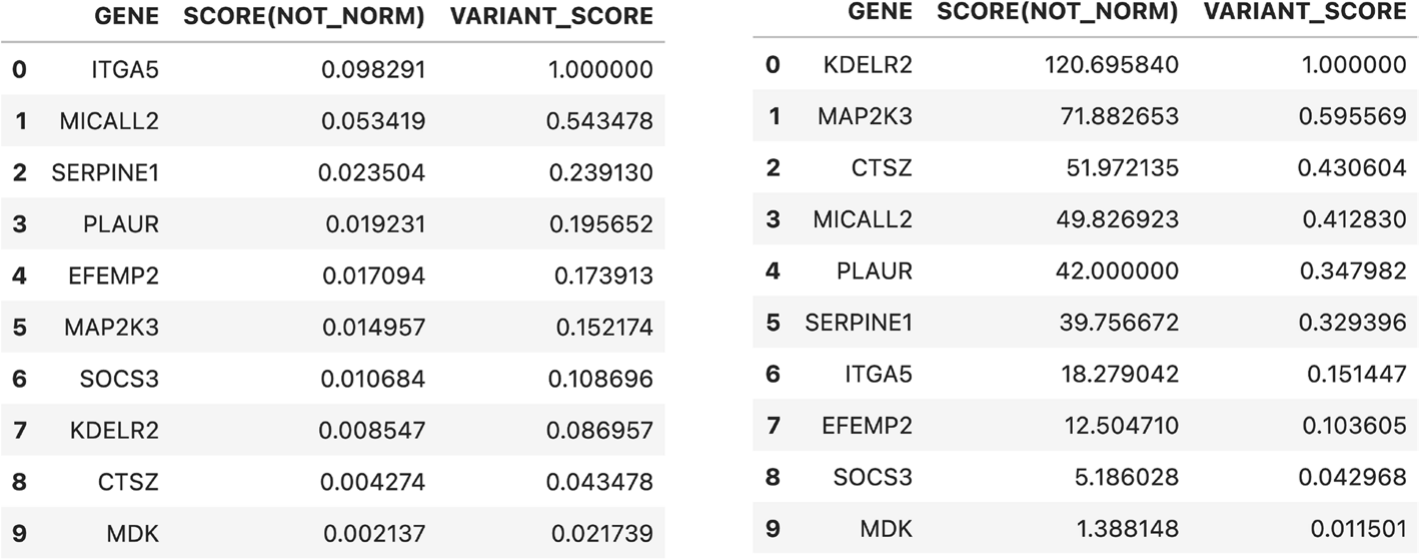
Candidate genes and their scores. On the left the scores for the tumor cohort; on the right the ones for the healthy cohort

By composing the two described operations, we can compute the scores for a list of candidate genes for the two considered cohorts. Assuming the following candidate genes [CTSZ, EFEMP2, ITGA5, KDELR2, MAP2K3, MDK, MICALL2, PLAUR, SERPINE1, SOCS3], a tumor cohort including all *skin cutaneous melanoma* patients with somatic mutations aligned to GRCh38, and a healthy cohort including all donors having germline variants aligned to GRCh38, using VarSum we can easily compute the gene scores and their rank normalization, shown in Fig. 7.

**Fig. 8:**
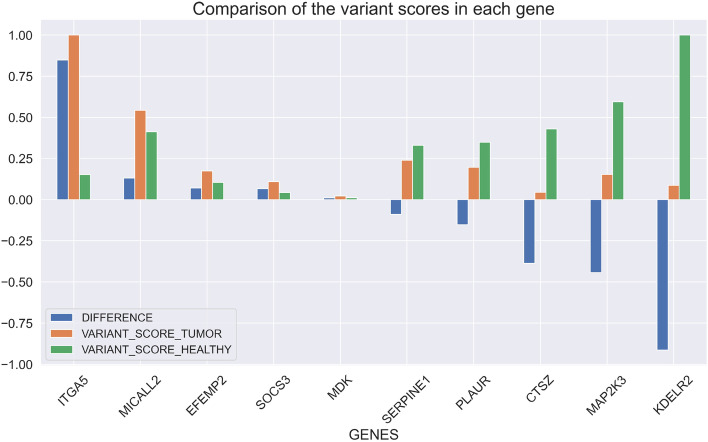
Candidate genes and their scores. On the left the scores for the tumor cohort; on the right the ones for the healthy cohort

As an evaluation metric for assessing the relevance of each candidate gene in the *skin cutaneous melanoma*, for every gene we compute the difference between its normalized scores in the tumor and in the healthy cohorts, obtaining the results shown in Fig. 8. Thus, among the selected candidate genes we identify the ITGA5 gene as the one most likely involved in the *skin cutaneous melanoma*. This finding is confirmed in recent studies [64].

## Discussion

We presented two separate implementation contributions, the first one being the integration of population variation datasets within a tertiary analysis repository, and the second one being a computational framework with an API to flexibly query the integrated datasets for sample-set extraction and population variant analysis.

Regarding the first contribution, we proposed to transform input datasets from the VCF classical format into a GDM-based one. These are two very different formats to represent genomic variants; the steps necessary to perform the conversion are many and quite complex overall. Also the size of the generated datasets is considerably greater than the original one, since the VCF format offers a greater compression ratio when describing variants that are common to a large set of individuals. However, conversely the GDM-based format makes the valuable VCF data and related metadata seamlessly integrated with all other heterogeneous genomic data from multiple sources publicly available in the META-BASE repository, including somatic mutations from TCGA, as well as comprehensively queryable and evaluable efficiently in the cloud thanks to the GMQL technology and its ecosystem of publicly available interfaces and tools.

For what concerns our second contribution, the provision of a computational framework with an API for flexible population variation querying, previously proposed strategies, which rely on pre-computed population statistics (e.g., gnomAD), limit the possibility to refine studied populations in depth. More sophisticated population characterisations are enabled by general genomic computing systems, such as GMQL, where a variety of data sources can be comprehensively queried using a dedicated language; yet, the learning barriers of the latter ones are substantial, due to the complexity of the systems and their ad-hoc query languages [7, 20, 65, 66]. Other approaches, offering practical user interfaces (e.g., Ensembl [17], or PGG.SNV [23]), do not provide APIs that are easy to be integrated within bioinformatic pipelines.

Conversely, VarSum, whose goal is to support the analysis of the genomic variation characteristics of a user-defined population, offers several advantages:

1. on the technological side, we claim a fast learning curve of the VarSum services and their parameters, and their easy integration within programming codes;
2. on the interoperability side, we have fully integrated the 1000 Genomes Project variant datasets within the GMQL and GenoSurf systems;
3. on the functional side, we have developed a framework and an API to support the aggregated analysis of DNA variants of a population that the user can arbitrarily define through fine-grained requirements over metadata and genomic region data of the considered datasets, also allowing comparing the frequency of DNA variants in any desired (sub)population.

We integrated variation data from 1KGP and TCGA, as well as gene annotations from GENCODE; more datasets of DNA variants or relevant genomic regions can be further integrated by seamlessly adding dedicated software classes extending the *Annotation Source* or *Variation Source* classes of VarSum. In particular, we plan to integrate additional data of other sequencing projects, such as the “1+ Million Genomes Project” [67] that started recently, as well as a broader set of genome annotation types, beyond gene annotations. Moreover, as the release of whole genome variation studies has recently raised privacy concerns [68], VarSum provides built-in support for ready-made privacy constraints (e.g., by refusing to answer the user’s request if the result involves data only from a small number of donors). Still, more complex privacy rules will be added per request and data-source, if needed. Even though VarSum scales well with the number of data sources, critical aspects emerge when ranking variants by frequency on large whole-genome data collections as 1KGP. The calculation of the most frequent/rarest variants requires scanning and grouping operations for each variant in the population of interest. For 1KGP data, this results into a number of variants increasing by 4.4 million per donor, corresponding to a response time of  9 seconds per individual^3^. To improve this performance, two main optimisations will be targeted as future work: offline pre-calculation of results (for typical queries that do not use genomic region data filters) and response caching.

## Conclusions

The 1000 Genomes Project is the most recent whole-genome sequencing initiative that publicly released a big collection of DNA variants and population data without any embargo. Similar or larger projects were started afterwards, especially in the form of large-scale genomic national initiatives [69]. Examples include *All of US* [70] by the National Institutes of Health (NIH) in the United States, the *100,000 Genomes Project* [5] (a United Kingdom Government project that is sequencing whole genomes from UK National Health Service patients), or deCODE Genetics [71], a private company that initiated the full sequencing of the Icelandic population. Assuming that these project results will be released publicly in the future, they will need to be considered within the scope of future public data integration efforts, giving a new and substantial boost to the potential of genomic data analysis. At that time, there will be even a stronger need for instruments for genomic data aggregation and querying such as the one here proposed, allowing free characterizations of populations from a genomic/evolutionary standpoint.

### Availability and requirements

- **Project name**: VarSum
- **Project home page**: http://www.gmql.eu/popstudy/
- **Operating system(s)**: Platform independent
- **Programming language**: Scala is the main programming language used for the data integration task, as it is the language of development of the META-BASE project. The data integration procedure involves also the use of XML files for defining configuration options as well as the output region file schema. Instead, both the software loading GDM region data files to the database and the VarSum API have been developed using Python as the main programming language, in combination with SQL for making database queries. VarSum also makes extensive use of the YAML [72] language for the API documentation.
- **Other requirements**: Java 1.7 or higher is required to run Metadata-Manager (https://github.com/DEIB-GECO/Metadata-Manager/). Instead, the database loading software and the VarSum API server need Python 3.7 to be installed. VarSum and the database loading software have separate software package dependencies, respectively listed inside a file named *requirements.txt*, as is usual in Python projects. One of such files is present in each project’s GitHub repository: at https://github.com/DEIB-GECO/VarSum for the API server, and at https://github.com/DEIB-GECO/geco_agent_loader for the database loading software. Additionally, a PostgreSQL database server running in the same machine is needed for the proper execution of the software hereby discussed.
- **License**: The software module developed for the integration of 1KGP data inside the META-BASE repository is part of the Metadata-Manager software package (https://github.com/DEIB-GECO/Metadata-Manager/) and is available under the same Apache-2.0 License. Likewise, the Apache-2.0 License applies to the database loading software (https://github.com/DEIB-GECO/geco_agent_loader). The VarSum software is released under the GNU General Public License (GPL) v3.0.
- **Any restrictions to use by non-academics**: The same restrictions provided by the GNU GPL License v3.0 apply to academic and non-academic use.

## Supplementary Information

**Additional file 1.** Example of translation from VCF into GDM format for genomic region data: This .xlsx (MS Excel) spreadsheet exemplifies the transformation of the original 1KGP mutations—expressed in VCF format—into GDM genomic regions. As a demonstrative example, some variants about chromosome X have been selected from the source data (in VCF format) and listed in the first table at the top of the file. The values of columns *#CHROM*, *POS*, *REF* and *ALT* appear as in the source. We removed the details that are unnecessary for the transformation from the column *INFO*. In the column *FORMAT* it is indicated exclusively the value “GT”, meaning that the next columns contain only the genotype of the samples (this and other conventions are expressed in the VCF specification document and in the header section of each VCF file). In multiallelic variants (examples *e*, *f*.1 and *f*.2), the genotype indicates with a number which of the alternative alleles in *ALT* is present in the corresponding samples (e.g., the number 2 means that the second variant is present); otherwise, it only assumes the values 0—mutation absent, or 1—the mutation is present. Additionally, the genotype indicates whether one or both chromosome copies contain the mutation and which one, i.e., the left one or the right one; the mutated alleles are normally separated by a pipe (“|”), if not otherwise specified in the header section; we do not know which chromosome copy is maternal or paternal, but as the 1KGP mutations are “phased”, we know that the “left chromosome” is the same in every mutation located in the same chromosome of the same donor. As in this example we have only one column after the *FORMAT* one, the mutations described are relative to only one sample, called “HG123456”. Actually, this sample does not exist in the source, but serves the purpose of demonstrating several mutation types that are found in the original data. The table reports six variants in VCF format, with the last one repeated two times to show how different values of genotype lead to a different translation (indeed, examples *f*.1 and *f*.2 differ only for the last column). Below in the same file, the same variants appear converted in GDM format. The transformation outputs the *chr*, *left*, *right*, *strand*, *AL1*, *AL2*, *ref*, *alt*, *mut_type* and *length* columns. The value of strand is positive in every mutation, as clarified by the 1KGP Consortium after the release of the data collections. Values of *AL1* and *AL2* express on which chromatid the mutation occur and depend on the value of the original genotype (column *HG123456*). The values of the other columns, namely *chr*, *left*, *right*, *ref*, *alt*, *mut_type* and *length*, are obtained from the variant original values after the split of multi-allelic variants, the transformation of the original position into 0-based coordinates, and the removal of repeated nucleotide bases from the original *REF* and *ALT* columns. In 0-based coordinates, a nucleotide base occupies the space between the coordinates *x* and *x* + 1. So, SNPs (examples *a* and *f*.2) are encoded as the replacement of *ref* at position between *left* and *right* with *alt*. Insertions (examples *c* and *f*.1) are described as the addition of the sequence of bases in *alt* at the position indicated in *left* and *right*, i.e., in between two nucleotide bases. Deletions (example *b*) are represented as the substitution of *ref* between positions *left* and *right* with an empty value (*alt* is indeed empty in this case). Finally, structural variants (examples *d* and *e*) such as copy number variations and large deletions have an empty *ref* because, according to the VCF specification document, the original column *REF* reports a nucleotide (called padding-base) that is located before the scope of the variant on the genome and is unnecessary in a 0-based representation. In this file, we reported only the columns relevant for the understanding of the transformation method regarding the mutation coordinates, reference and alternative alleles. Actually, in addition to the ones reported in the second table, the transformation adds some more columns, called as the attributes in the original *INFO* column to capture a selection of the attributes present in the original file.

**Additional file 2.** Example of transformed metadata: In this .xlsx (MS Excel) file, we list all the output metadata categories generated for each sample from the transformation of the 1KGP input datasets. The output metadata include information collected from all the four 1KGP metadata files considered. Some categories are not reported in the source metadata files—they are identified by the label *manually_curated__...*—and were added by the developed pipeline to store technical details (e.g., download date, the md5 hash of the source file, file size, etc.) and information derived from the knowledge of the source, such as the species, the processing pipeline used in the source and the health status. For every information category, the table reports a possible value. The third column (*cardinality* > 1) tells whether the same key can appear multiple times in the output GDM metadata file. This is used to represent multi-valued metadata categories; for example, in a GDM metadata file, the key *manually_curated__chromosome* appears once for every chromosome mutated by the variants of the sample.

## Data Availability

The documentation and the source code of META-BASE and its 1KGP extension are available at https://github.com/DEIB-GECO/Metadata-Manager/. The source code of the software loading the GDM region data to the database is available at https://github.com/DEIB-GECO/geco_agent_loader. VarSum and its API are hosted at http://www.gmql.eu/popstudy/, where it is also possible to try the API endpoint calls with one of the prebuilt examples or with user-input parameters. The software of VarSum (written in Python) is freely accessible at the GitHub repository https://github.com/DEIB-GECO/VarSum. Documentation, ready-to-run examples in the form of IPython Notebooks and two explanatory videos are available at both locations. The datasets produced (1KGP) or referenced throughout this study (TCGA, GENCODE gene annotations, and others) are publicly available in the META-BASE repository at http://www.gmql.eu/ and through the APIs of GMQL (REST, Python and R/Bioconductor, see http://www.bioinformatics.deib.polimi.it/geco/?try).

## Abbreviations

1KGP: 1000 Genomes Project
API: Application Programming Interface
BED: Browser Extensible Data
EVS: Exome Variant Server
GCM: Genomic Conceptual Model
GDC: Genomic Data Commons
GDM: Genomic Data Model
GMQL: GenoMetric Query Language
gnomAD: Genome Aggregation Database
GPL: General Public License
HTTP: Hypertext Transfer Protocol
IGIB: Institute of Genomics & Integrative Biology
IGSR: International Genome Sampling Resource
JSON: JavaScript Object Notation
MNP: multi-nucleotide polymorphism
NGS: Next-Generation Sequencing
NIH: National Institutes of Health
rDNA: ribosomal DNA
REST: Representational state transfer
SAGE: South Asian whole genomes and exomes
SNP: single nucleotide polymorphism
SQL: Structured Query Language
TCGA: The Cancer Genome Atlas Program
URI: Uniform Resource Identifier
VCF: Variant Call Format
XML: Extensible Markup Language

## Footnotes

1 The FTP locations of the hg19 and GRCh38 datasets are reported at [48] and [49], respectively.

2 Metadata about the indexes of the alignment files [50]; information on the 26 populations participating to the project [51]; characteristics of the individuals donating the samples [52]; sequencing strategies [53].

3 Such performance corresponds to the tests carried on the same machine used for the integration of 1KGP data into the META-BASE repository (see Result section).
